# Cationic biocide susceptibility and tolerance-associated genes in clinical, environmental, and commensal *Enterococcus* isolates

**DOI:** 10.1038/s41598-026-51663-z

**Published:** 2026-05-13

**Authors:** Maha M. Eldahshan, Asmaa K. Amer, Doaa E. Genena, Basma Mahmoud Awad Allah Nasr, Maha Mostafa Hana, Asmaa E. Mohamed

**Affiliations:** 1 Department of Medical Microbiology & Immunology, Faculty of Medicine, Menoufia University, Shebin El Kom, Egypt; 2 Department of Clinical Pathology, Faculty of Medicine, Menoufia University, Shebin Al- Kom, Egypt; 3 Department of Critical Care Medicine, Faculty of Medicine, Menoufia University, Shebin Al- Kom, Egypt; 4 Department of Tropical Medicine, Faculty of Medicine, Menoufia University, Shebin Al- Kom, Egypt; 5 Department of Clinical Microbiology &Immunology, National Liver Institute, Menoufia University, Shebin Al- Kom, Egypt

**Keywords:** *Enterococci*, Benzalkonium chloride, Chlorhexidine digluconate, *EfrAB*, *EmeA*, Antibiotics, Biofilm, Diseases, Microbiology

## Abstract

*Enterococcus* spp. are important etiological agents of both healthcare associated and community-acquired infections, necessitating effective antisepsis and disinfection strategies to limit their transmission. This study aimed to evaluate the susceptibility of *Enterococcus* isolates from clinical, environmental, and commensal sources to benzalkonium chloride (BCC) and chlorhexidine digluconate (CHX), and to investigate their association with selected efflux-associated genes. A total of 520 specimens were collected from clinical settings, hospital environments, and faecal samples obtained from healthy individuals. From these, 120 *Enterococcus* isolates were recovered and subjected to antimicrobial susceptibility testing. Minimum inhibitory concentrations (MICs) for CHX and BCC were determined using an agar dilution method under standardized laboratory conditions. Biofilm formation was quantified using a microtiter plate assay, and the efflux pump genes *emeA*,* efrA*, and *efrB* were detected by PCR. Clinical isolates demonstrated significantly higher resistance to gentamicin and ciprofloxacin, along with reduced susceptibility to CHX and BCC, as reflected by higher MIC₅₀ and MIC₉₀ values. Biofilm formation was observed in 66.7% of isolates and was significantly more prevalent among clinical strains. Isolates harboring efflux pump genes exhibited higher MIC₉₀ values for both biocides, and *efrAB* and *emeA* were strongly associated with gentamicin resistance. Overall, clinical *Enterococcus* isolates exhibited higher antimicrobial resistance, enhanced biofilm-forming capacity, and reduced susceptibility to CHX and BCC compared with environmental and commensal isolates. These findings suggest a potential role of efflux-mediated mechanisms in reduced biocide susceptibility and antibiotic resistance.

**Supplementary Information:**

The online version contains supplementary material available at 10.1038/s41598-026-51663-z.

## Introduction

*Enterococci* are listed by the World Health Organization (WHO) as priority pathogens and are frequently implicated in hospital-acquired infections (HAIs). Their clinical importance continues to rise due to the emergence of strains exhibiting resistance to multiple antimicrobial agents^1^. Among more than 60 recognized *Enterococcus* species, *Enterococcus faecalis* and *Enterococcus faecium* are the primary contributors to human opportunistic infections and are also dominant members of the human intestinal microbiota^2^.

Transmission of *Enterococcus* spp. within healthcare environments occurs readily through faecal contamination of the hands of patients, healthcare workers, and visitors, as well as via contaminated medical equipment and inanimate surfaces^3^. Consequently, effective antiseptic and disinfection strategies are essential to interrupt.

transmission pathways, limit environmental dissemination, and prevent potentially life-threatening multi-drug-resistant (MDR) infections^4^.

Cationic biocides (CBs) constitute a major class of broad-spectrum antimicrobial agents widely incorporated into disinfectants, antiseptics, and preservatives^4,5^. Their use spans clinical and domestic environments, agriculture, the food industry, and other sectors, with applications dating back to the 1930s^6^. Among these agents, quaternary ammonium compounds (QACs) and biguanides have received particular attention due to increasing interest in *Enterococcal* susceptibility patterns^7^.

Benzalkonium chloride (BCC), a QAC, and chlorhexidine digluconate (CHX), a water-soluble biguanide, are among the most widely used CBs in hospitals, households, farms, and food production facilities. BCC exerts its antimicrobial activity primarily through the denaturation of cytoplasmic membrane proteins, whereas CHX disrupts membrane integrity by inducing protein denaturation and inhibiting membrane-associated enzymes, ultimately impairing cell growth^8^.

Although biocides provide substantial benefits, their extensive and often indiscriminate use contributes to environmental contamination and increases the likelihood of microbial exposure to sub-lethal concentrations. Such conditions facilitate the emergence of reduced biocide susceptibility. Furthermore, genomic mutations and acquired resistance determinants associated with biocide tolerance are increasingly linked to antibiotic cross-resistance in bacterial pathogens^9^.

In *Enterococci*, several multidrug efflux pumps have been identified. Members of the major facilitator superfamily (MFS), including *emeA*, *qacA*, and *qacB*, contribute to resistance against aminoglycosides, fluoroquinolones, and reduced susceptibility to BCC. Additional efflux systems *qacE*,* qacF*,* qacG*,* qacZ*, and smr belong to the small multidrug resistance (SMR) family, while e*frA* and *efrB* represent ATP-binding cassette (ABC) transporters^8^. Cross-resistance to antibiotics and biocides may arise through shared mechanisms such as efflux pump induction or the horizontal transfer of resistance genes carried on mobile genetic elements^10^.

Biocides may also promote biofilm formation under certain conditions. Sub-lethal biocide exposure can act as an environmental stressor, stimulating bacteria to form biofilms as a protective response^11^. Biofilms enhance collective resistance by shielding cells from antimicrobial agents. Additionally, biocides may upregulate efflux pump expression, contributing both to biocide expulsion and to enhanced adhesion and biofilm development^12^. Alterations in membrane composition following biocide exposure may further facilitate bacterial attachment to surfaces^13^.

The objectives of this study were to determine the (MICs) of chlorhexidine (CHX) and benzalkonium chloride (BCC) against *Enterococcus* spp. isolates; to evaluate potential cross-resistance between these biocides and selected antibiotics (ciprofloxacin, vancomycin, and gentamicin); to assess biofilm formation and its association with biocide susceptibility, including the potential influence of sub-inhibitory concentrations; and to detect selected biocide-associated resistance genes (emeA, efrA, and efrB) using PC.

### Patients and methods

#### Study design and ethical considerations

This cross-sectional study included a comprehensive collection of 520 isolates obtained between 2025 and 2026. Clinical specimens were collected from patients admitted to various hospital wards (medical & tropical, surgical, pediatrics, and Obstetrics & Gynecology wards) and Intensive Care Units (ICUs), as well as from individuals attending outpatient clinics at Menoufia University Hospitals (MUHs) and the National Liver Institute (NLI). Environmental samples were obtained from surfaces within different hospital wards and ICUs, and additional samples were collected from healthy carriers. Ethical approval for the study was granted by the Ethics Committee of the Faculty of Medicine, Menoufia University (IRB: 00768/2025).

#### Sample collection and identification of ente*rococcus* spp

Of the 520 collected samples, 120 isolates were identified as *Enterococcus* spp. and categorized as clinical, environmental, and healthy-volunteer isolates. Clinical specimens included urine, blood, sputum, wound exudates, surgical drain fluids, burn wound swabs, and ascitic fluid. Environmental isolates were obtained from surfaces in hospital wards and ICUs, while non-clinical isolates were recovered from the faeces of healthy volunteers.

Specimens were cultured on standard bacteriological media (Oxoid, England), and species identification was performed using the Vitek-2 Compact System (GP-REF 21342). Isolates were preserved in 30% glycerol broth at − 80 °C for subsequent analyses.

### Antimicrobial susceptibility testing

#### Antibiotics

Antimicrobial susceptibility of *Enterococcus faecalis* and *Enterococcus faecium* isolates was determined using the VITEK^®^ 2 Compact system (bioMérieux, France) with AST-P592 cards, according to the manufacturer’s instructions. The panel included ampicillin, high-level gentamicin, high-level streptomycin, ciprofloxacin, erythromycin, vancomycin, linezolid, teicoplanin, tetracycline, and tigecycline. Minimum inhibitory concentrations (MICs) were automatically generated by the system and interpreted in accordance with the Clinical and Laboratory Standards Institute (CLSI) guidelines (2025)^14^.For quality control, *E. faecalis* ATCC 29,212 was used.

#### Cationic biocides

MICs for BCC and CHX were determined using the agar dilution method. BCC (LOBA Chemical, India; 50 g/100 mL) was serially diluted in Mueller–Hinton agar to obtain concentrations ranging from 0.125 to 1024 µg/mL. CHX-HCl (Hexitol, Egypt; 125 mg/100 mL) was diluted similarly. Plates were incubated at 37 °C, and the MIC was determined as the lowest concentration of biocide at which no visible colony growth of the studied *Enterococcus* isolate was observed. Results were presented as MIC₅₀, indicating the concentration at which 50% or more of the tested strains were inhibited, and MIC90, indicating the concentration at which 90% or more of the tested strains were inhibited.

#### Biofilm formation assay

Biofilm formation was assessed using a microtiter plate assay. Strains were adjusted to 0.5 McFarland in physiological saline and inoculated into Tryptic Soy Broth (TSB) supplemented with 1% glucose. A 20 µL inoculum was added to 180 µL of supplemented TSB in flat-bottomed microtiter plates and incubated at 37 °C for 24 h. Wells were washed three times with phosphate-buffered saline (PBS), air-dried overnight, and stained with 1% crystal violet for 15 min. After rinsing and drying, 200 µL of 96% ethanol was added, and absorbance was measured at 570 nm using a microplate reader (Azure Biosystems, Dublin, CA, USA). All assays were performed in triplicate, with TSB-glucose serving as the negative control. Biofilm formation was categorized based on optical density (OD) values as follows: non-biofilm producers (OD ≤ ODc), weak (ODc < OD ≤ 2×ODc), moderate (2×ODc < OD ≤ 4×ODc), and strong biofilm producers (OD > 4×ODc), where ODc represents the cutoff value as described by Gorski et al.^15^.

#### Molecular detection of efflux pump genes associated with cationic biocide susceptibility

All isolates were screened for the efflux pump genes *emeA*, *efrA*, and *efrB* using polymerase chain reaction (PCR). Genomic DNA was extracted using the Thermo Fisher GeneJET purification kit and stored at − 20 °C. PCR amplification was performed using a T Professional Thermocycler (Biometra, Germany) with gene-specific primers. The optimized PCR conditions are summarized in Table 1^8^. Amplified products were separated on 1.5% agarose gel at 100 V for one hour and visualized under a UV transilluminator. Expected amplicon sizes were 128 bp (*emeA*), 224 bp (*efrA*), and 211 bp (*efrB*) **(Supplementary material—Figures S1**,** S2)**.

**Table 1:** Primer sequence of target genes.

Genes	Primers	Amplicon size (bp)	Reference
***emeA***	F-AGCCCAAGCGAAAAGCGGTTT R-CCATCGCTTTCGGACGTTCA	128	^8^
***efrA***	F-TTGGCTTTATGACGCCAGT R-ATGCGCGTATTACCCGCAA	224	
***efrB***	F-TAGTGATGATGTTCTTAATCAA R-ATTGACTTGTTTAAAGCCTTCA	211	

### Statistical analysis

All collected data were organized and analyzed using SPSS version 26. Descriptive and analytical statistical methods were used, and the results were presented numerically and graphically. The Chi-squared test (χ2) was used for categorical variables. A p-value ≤ 0.05 was considered statistically significant. Spearman’s correlation and the chi-squared test were used to measure the strength and direction of the association between biofilm-formation ability and susceptibility to biocides.

## Results

### Prevalence of *enterococcus* spp. and distribution of specimens

**Fig. 1:**
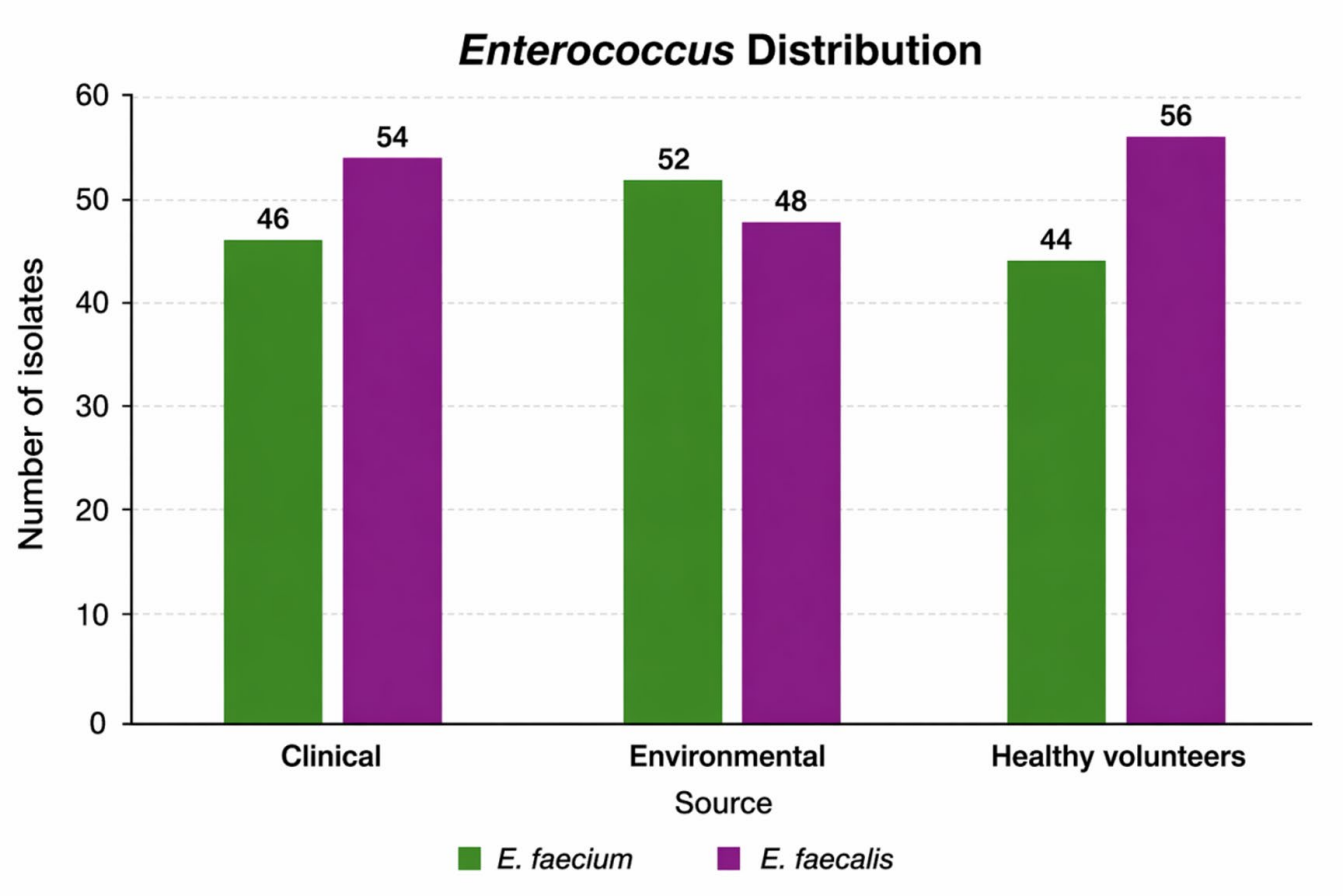
Frequency distribution of *Enterococcus* isolates among the isolated specimens.

*Enterococcus* species accounted for 23.1% of all recovered isolates. *E. faecalis* was the predominant species (52.5%), followed by *E. faecium* (47.5%). Among clinical isolates (*n* = 60, 50%), *E. faecalis* and *E. faecium* represented 53.3% and 46.7%, respectively. In environmental samples (*n* = 33, 27.5%), the distribution was 48.5% and 51.5%, respectively, while isolates from healthy volunteers (*n* = 27. 22.5%) comprised 55.6% *E. faecalis* and 44.4% *E. faecium* **(**Fig. 1**).**

Urine samples yielded the highest proportion of isolates (50%), followed by blood (30.8%). while ascetic fluid and wound swabs yielded the lowest proportion (3.3%, and 2.5%), respectively. *Enterococcus* spp. were also detected alongside other bacterial isolates, including *Escherichia coli*,* Staphylococcus aureus*,* Klebsiella* spp., and coagulase-negative *staphylococci*.

### Antimicrobial susceptibility testing

The Vitek-2 Compact System demonstrated high susceptibility to tigecycline (100%), teicoplanin (83.3%), linezolid (87.5%), and vancomycin (77.5%). Resistance was observed for tetracycline (58.3%) and ciprofloxacin (51.7%). Streptomycin (54.2%) and high-level gentamicin (43.3%) also showed notable resistance rates **(Supplementary material—Table S1).**

### Susceptibility of enterococcus spp. to ciprofloxacin, vancomycin, and gentamicin according to origin

Both *E. faecalis* and *E. faecium* exhibited significantly higher gentamicin resistance in clinical isolates (81.8% and 86.6%) compared with environmental and healthy-carrier isolates (*p* < 0.001). Ciprofloxacin resistance in *E. faecium* was also significantly elevated in clinical isolates (65.8%). In *E. faecalis*, resistance levels were moderate across all sources without significant variation (*p* > 0.5). Vancomycin resistance was highest in clinical isolates (66.7%), though not statistically significant for either species **(**Table 2**).**

**Table 2:** Antibiotic susceptibility of *Enterococcus* spp. to ciprofloxacin, vancomycin, and gentamicin across different isolation sources.

Organisms	Antibiotics		Clinical (*n* = 32)		Surfaces (*n* = 16)		Healthy carrier (*n* = 15)		p*
			No.	%	No.	%	No.	%	
***E.faecalis***	**CIP**	**R**	14	58.3	6	25	4	16.7	**0.53**
		**S**	18	46.2	10	25.6	11	28.2	
	**VAN**	**R**	3	33.3	1	11.1	5	55.6	**0.518**
		**S**	29	53.7	15	27.8	10	18.5	
	**GEN**	**R**	18	81.8	1	4.5	3	13.6	0.001*
		**S**	14	34.1	15	36.6	12	29.3	
			**Clinical** (*n* = 28)		**Surfaces** (*n* = 17)		Healthy carrier (*n* = 12)		
			**N %**		**N %**		**N %**		
***E.faecium***	**CIP**	**R**	25	65.8	8	21.2	5	13.2	0.001*
		**S**	3	15.8	9	47.7	7	36.8	
	**VAN**	**R**	12	66.7	2	11.1	4	22.2	**0.0938**
		**S**	16	41	15	38.5	8	20.5	
	**GEN**	**R**	26	86.6	2	6.7	2	6.7	< 0.001*
		**S**	2	7.4	15	55.6	10	37	

p*: p value for comparing between the three isolation sources regarding antibiotics susceptibility.

*: Statistically significant at *p* ≤ 0.05.

### MIC distribution of chlorhexidine digluconate (CHX)

CHX MICs ranged from 0.5 to 16 µg/mL. Clinical isolates of *E. faecalis* and *E. faecium* showed the highest proportion of elevated MICs compared to environmental and healthy-carrier isolates, with 62.5% and 75% of isolates, respectively, exhibiting MICs of 8 µg/mL indicating reduced susceptibility and the difference was statistically significant. MIC₅₀ and MIC₉₀ values for clinical isolates were identical (8 µg/mL) for both species. Environmental and healthy-carrier isolates demonstrated lower MIC₅₀ values (4 µg/mL), indicating greater susceptibility **(**Table 3**).**

**Table 3:** MIC profiles of chlorhexidine digluconate for *E. faecalis* and *E. faecium* based on the isolation source.

SPP.	Sources	Chlorohexidine digluconate MIC (µg/mL)													
		0.5		1		2		4		8		16		MIC50 (µg/mL)	MIC90 (µg/mL)
		*N*	%	*N*	%	*N*	%	*N*	%	*N*	%	*N*	%		
***E.faecalis ***	**Clinical** *N* = 32	**0 (0.0)**		**0 (0.0)**		**3 (9.4)**		**9 (28.1)**		**20 (62.5)**		**0 (0.0)**		**8**	**8**
	**Surfaces** *N* = 16	**1(6.25)**		**0 (0.0)**		**2(12.5)**		**8(50)**		**5 (31.25)**		**0 (0.0)**		**4**	**8**
	**Healthy carriers** *N* = 15	**0 (0.0)**		**3(20)**		**4(26.7)**		**5(30)**		**3(20)**		**0 (0.0)**		**4**	**8**
	Total *N* = 63	**1 (1.6)**		**3 (4.8)**		**9(14.3)**		**22(34.9)**		**28 (44.4)**		**0 (0.0)**		**4**	**8**
P1 < 0.05 p2 < 0.05^*^ **p3 < 0.05**															
***E.faecium***	**Clinical** *N* = 28	**0 (0.0)**		**0 (0.0)**		**2(7.1)**		**4(14.3)**		**21(75)**		**1 (3.6)**		**8**	**8**
	**Surfaces** *N* = 17	**0 (0.0)**		**0 (0.0)**		**4(23.5)**		**9(52.9)**		**4 (23.5)**		**0 (0.0)**		**4**	**8**
	**Healthy carrier** *N* = 12	**0 (0.0)**		**2 (16.7)**		**3 (25)**		**5 (41.7)**		**2 (16.7)**		**0 (0.0)**		**4**	**8**
	Total *N* = 57	**0 (0.0)**		**2(3.5)**		**9(15.8)**		**18(31.6)**		**27(47.4)**		**1(1.8)**		**4**	**8**

**p**_**1**_<0.001^*^ p2 < 0.001^*^ **p3 =** 1.0, P1: p value for comparing between clinical vs. surfaces regarding CHX MIC values. P2: p value for comparing between clinical vs. healthy Carriers regarding CHX MIC values. P3: p value for comparing between surfaces vs. healthy Carriers regarding CHX MIC values. *: Statistically significant at *p* ≤ 0.05.

### MIC distribution of benzalkonium chloride (BCC)

BCC MICs ranged from 2 to 32 µg/mL. Clinical isolates of *E. faecalis* and *E. faecium* demonstrated a higher proportion of isolates with MIC values of 16 µg/mL (56.25% and 53.6%, respectively) compared to isolates from environmental surfaces and healthy carriers. This difference was statistically significant for *E. faecium* (*p* < 0.05), indicating a higher distribution of elevated MICs among clinical isolates relative to the other sources **(**Table 4**).**

**Table 4:** MIC profiles of benzalkonium chloride for *E. faecalis* and *E. faecium* based on the isolation source.

Organisms	Sources	Benzalkonium chloride MIC (µg/mL)						
		2	4	8	16	32	MIC50	MIC90
		NO(%)	NO(%)	NO(%)	NO(%)	NO(%)	NO(%)	NO(%)
***E.faecalis ***	**Clinical** *N* = 32	2(6.25)	3(9.4)	9(28.1)	18(56.25)	**0 (0.0)**	**16**	**16**
	**Surfaces** *N* = 16	**0 (0.0)**	**2**(12.5)	**6**(37.5)	**8**(50)	**0 (0.0)**	**8**	**16**
	**Healthy carrier** *N* = 15	**1**(6.7)	**3**(20)	**5**(33.3)	**6**(40)	**0 (0.0)**	**4**	**16**
	Total *N* = 63	**3**(4.8)	**8**(12.7)	**21**(33.3)	**31**(49.2)	**0 (0.0)**	**8**	**16**
**P1 < 0.05 p2 < 0.05 p3 < 0.05**								
***E.faecium***	**Clinical** *N* = 28	**0 (0.0)**	**5**(17.9)	6(21.4)	**15**(53.6)	2(7.1)	16	16
	**Surfaces** *N* = 17	**2**(11.8)	**4**(23.5)	**7**(41.2)	**4**(23.5)	**0 (0.0)**	8	16
	**Healthy carrier** *N* = 12	**1**(8.3)	**2**(16.7)	**3**(25)	**5**(41.7)	1(8.3)	16	16
	Total *N* = 57	**3**(5.3)	**11**(19.3)	**18**(31.6)	**22**(38.6)	3(5.3)	16	16

P1 = 0.0297* p2 < 0.05 p3 < 0.05, P1: p value for comparing between clinical vs. surfaces regarding BCC MIC values. P2: p value for comparing between clinical vs. healthy carriers regarding BCC MIC values. P3: p value for comparing between surfaces vs. healthy carriers regarding BCC MIC values. *: Statistically significant at *p* ≤ 0.05.

### Relationship between biocide MICs and antibiotic resistance

under these experimental conditions, we found that in both species, isolates resistant to ciprofloxacin, vancomycin, and gentamicin consistently demonstrated higher MIC₅₀ and MIC₉₀ values (8, 16, and 32 µg/mL) for CHX and BCC compared with susceptible isolates. Exceptions included vancomycin-resistant *E. faecalis* for BCC (equal MIC₅₀/MIC₉₀ of 8 µg/mL in both groups) and gentamicin-resistant *E. faecium* for CHX (equal MIC₅₀/MIC₉₀ of 4 µg/mL) as illustrated in **(Supplementary material—Table S2).**

### Biofilm formation among enterococcus isolate

Biofilm formation was detected in 80 isolates (66.7%). *E. faecalis* exhibited a higher prevalence (71.4%, *n* = 45) than *E. faecium* (61.4%, *n* = 35). A pronounced and statistically significant relationship was identified between the isolation sources and the biofilm-forming capacity of both *E. faecalis* and *E. faecium* (*p* < 0.001 for each species). Clinical isolates showed the highest biofilm-forming capacity, with 96.9% of *Enterococcus faecalis* and 75% of *Enterococcus faecium* producing biofilms. Among these, 68.75% and 60.7%, respectively, were classified as strong biofilm producers (Category IV). In contrast, environmental isolates were predominantly moderate or weak biofilm producers, while isolates from healthy volunteers exhibited the lowest biofilm-forming capacity, with a higher proportion of non- or weak biofilm producers. **(Supplementary material—Table S3).**

### Association between biofilm strength and CHX/BCC MICs

For *E. faecalis* (*n* = 45), CHX MICs showed a weak negative correlation with biofilm strength (rs = − 0.114, *p* = 0.754), indicating no significant association. BCC MICs demonstrated a moderate negative correlation (rs = − 0.646, *p* = 0.044), suggesting that higher MICs were associated with reduced biofilm formation and that strong biofilm producers may be more susceptible to BCC.

For *E. faecium* (*n* = 35), CHX MICs showed a very weak positive correlation (rs = 0.078, *p* = 0.830), while BCC MICs showed a moderate negative correlation (rs = − 0.590, *p* = 0.072), indicating a trend toward increased BCC susceptibility among strong biofilm producers **(**Table 5**).**

**Table 5:** Correlation of biofilm-forming strength (grades II, III, IV) in *E. faecalis* and *E. faecium* with chlorhexidine digluconate and benzalkonium chloride MICs.

Biofilm-Forming Strength (II, III, IV)	CHX MICs		BCC MICs	
	*r*_s_	*P*	*r*_s_	*P*∗
E.faecalis (*n*=45)	−0.114	0.754	−0.646	0.044*
E.faecium (*n*=35)	0.078	0.83	−0.59	0.072

**r**_**s**_: Spearman coefficient. *: Statistically significant at *p* ≤ 0.05.

### Frequency distribution of efflux-pump genes associated with biocide susceptibility among en*terococcus* isolates

The *efrAB* gene was detected in 54% of *E. faecalis* and 53% of *E. faecium* isolates (overall 50.8%), with no significant difference between species (*p* = 0.47). The *emeA* gene was present in 39.7% of *E. faecalis* and 28% of *E. faecium* isolates (overall 34.2%), also without significant difference (*p* = 0.18). Co-existence of both genes occurred in 27% of *E. faecalis* and 17.5% of *E. faecium* isolates (overall 22.5%) (*p* = 0.216) as shown in **Supplementary material—Tables S4.**

### Distribution of efflux-pump genes according to origin of isolation of enterococci and their association with biocide MICs

A statistically significant difference was observed in the distribution of *efrAB* among the studied groups (*p* < 0.001), with the highest prevalence detected in clinical isolates compared to environmental and healthy carrier groups. In contrast, *emeA* showed no significant variation across the groups (*p* = 0.56), indicating a relatively uniform distribution. Notably, the co-existence of *efrAB* and *emeA* was significantly more frequent in clinical isolates (*p* < 0.001), suggesting a possible association with increased antimicrobial resistance in clinical settings **(**Table 6**).** No significant associations were observed between CHX or BCC MIC values and the presence of *efrAB* or *emeA* genes (*p* > 0.05). However, isolates harboring these efflux pump genes generally exhibited higher MIC values, although this trend did not reach statistical significance **(Supplementary material—Tables S5)**.

**Table 6:** Distribution of efflux pump genes among clinical, environmental, and healthy carriers *Enterococcus* isolates.

Variable	Clinical (*n* = 60)		Environmental (*n* = 33)		Healthy carrier (*n* = 27)				Total (*n* = 120)		*p*∗
	No.	%	No.	%	No.			%	No.	%	
	EfrAB										
**Present** **Absent**	40 20	65.6 34.4	12 21	36.4 63.6	7 20			25.9 74.1	61 59	50.8 49.2	< 0.001*
	***EmeA***										
**Present** **Absent**	22 38	36.7 63.3	10 23	30.3 69.7	9 18			33.3 66.7	41 79	34.2 65.8	0.56
	**Co-existence of** ***efrAB+emeA***										
**Present** **Absent**	20 40	33.3 66.7	4 29	8.3 91.7	1 26			3.7 96.3	25 95	20.8 79.2	< 0.001*

P*: p value for comparing between clinical and non-clinical *Enterococcus* isolates regarding efflux pump genes. ∗Statistically signifcant at *p* ≤ 0.05.

### Efflux-pump gene profiles and biocide MIC_90_ values

**Fig. 2:**
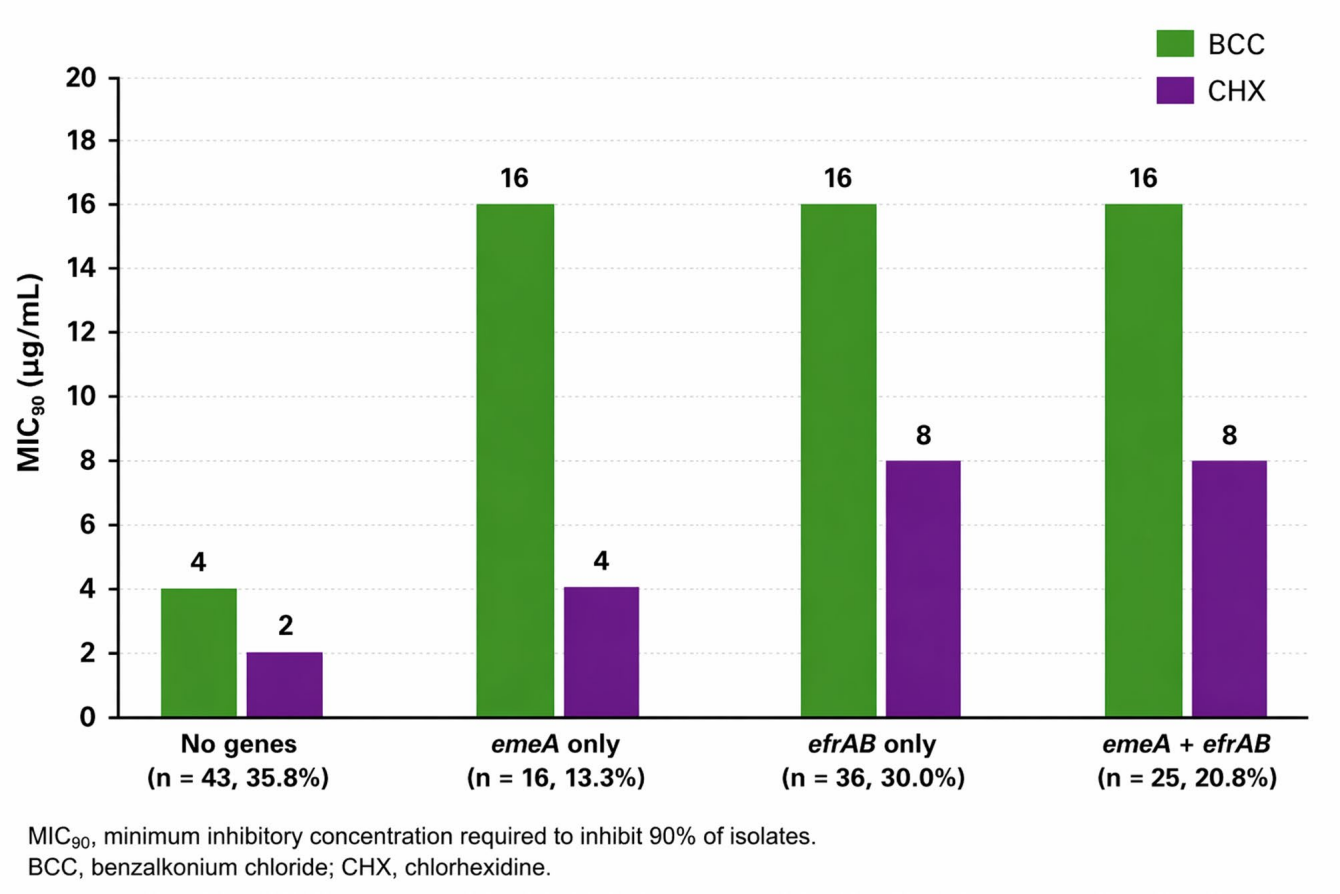
Effect of efflux pump gene profiles (*emeA*, *efrAB*) on biocide MIC<Subscript>90</Subscript> values in *Enterococcus* isolates.

*Enterococcus* isolates (*N* = 120) were categorized into four mutually exclusive groups based on efflux pump gene profiles: no genes (*n* = 43, 35.8%), *emeA* only (*n* = 16, 13.3%), *efrAB* only (*n* = 36, 30.0%), and co-existence of *emeA* and *efrAB* (*n* = 25, 20.8%). Isolates harboring either *emeA* or *efrAB*, alone or in combination, exhibited higher MIC₉₀ values for BCC and CHX compared with isolates lacking both genes **(**Fig. 2**)**. These findings underscore the potential contribution of efflux-pump genes to biocide resistance.

### Distribution of efflux-pump genes among antibiotic-resistant isolates

A strong and statistically significant association was observed between efflux gene carriage and gentamicin resistance. *EfrAB*-positive isolates showed markedly higher resistance (86.5%) than negative isolates (13.5%) (*p* < 0.001). *EmeA*-positive isolates also demonstrated significantly higher gentamicin resistance (53.8% vs. 19%; *p* = 0.00007). No significant associations were found between gene presence and resistance to ciprofloxacin or vancomycin **(**Table 7**).**

**Table 7:** Correlation of ciprofloxacin, vancomycin, and gentamicin resistance patterns with *efrAB* and *emeA* gene carriage in *Enterococcus* isolates.

Genotypes	CIP susceptibility patterns						p*
	Resistant (*n* = 62)		Susceptible (*n* = 58)		Total (*n* = 120)		
	No.	%	No.	%	No.	%	
***EfrAB*** Present Absent	35 27	56.5 43.5	26 32	44.8 55.2	61 59	50.8 49.2	0.2
***EmeA*** Present Absent	23 39	37.1 62.9	18 40	31 69	41 79	34.2 65.8	0.48
	**VAN susceptibility patterns**						
	**Resistant** **(n = 27)**		**Susceptible** **(n = 93)**		**Total** **(n = 120)**		
***EfrAB*** Present Absent	12 15	44.4 55.6	49 44	52.7 47.3	61 59	50.8 49.2	0.45
***EmeA*** Present Absent	10 17	37 63	31 62	33.3 66.7	41 79	34.2 65.8	0.72
	**GEN susceptibility patterns**						
	**Resistant** **(n = 52)**		**Susceptible** **(n = 68)**		**Total** **(n = 120)**		
***EfrAB*** Present Absent	45 7	86.5 13.5	16 52	23.5 76.5	61 59	50.8 49.2	< 0.001*
***EmeA*** Present Absent	28 24	53.8 46.2	13 55	19 81	41 79	34.2 65.8	< 0.001*

P*: p value for comparing between antibiotics susceptiblity regarding efflux pump genes carriage. *: Statistically significant at *p* ≤ 0.05.

## Discussion

*E.faecalis* and *E.faecium* remain prominent causative agents of hospital-acquired infections worldwide and continue to present significant clinical and epidemiological concerns. Their capacity to persist in environmental niches particularly those containing organic material has been linked to reduced susceptibility to both antibiotics and widely used biocidal agents^15^. Biocides such as antiseptics, disinfectants, sterilants, and preservatives are routinely applied in healthcare and community settings to limit microbial contamination^16,17^.

In this study, 120 *Enterococcus* isolates were examined, comprising 52.5% *E. faecalis* and 47.5% *E. faecium.* This species distribution is comparable to reports from Egypt^18,19^ and Iran^20^, where similar proportions have been observed.

The isolates were obtained from clinical samples (50%), environmental sources (27.5%), and faeces of healthy individuals (22.5%). A comparable distribution of isolates from clinical and non-clinical sources has been reported by Sobhanipoor et al^8^., who documented 54 clinical and 50 non-clinical isolates, including those from faecal samples. Additionally, another study^5^ included isolates from all three categories (clinical, environmental, and faecal sources), supporting the diversity of sampling approaches used in *Enterococcus* research.

Urine samples represented the most frequent source of *Enterococcal* isolation (50%), followed by blood (30.8%), with fewer isolates recovered from surgical drains, sputum, and ascitic fluid. These findings are consistent with previous reports from Egypt^18,21^.

Antimicrobial susceptibility testing revealed high sensitivity to tigecycline (100%), teicoplanin (83.3%), and linezolid (87.5%), in agreement with the results of Elbrolosy et al^18^.. Comparable susceptibility patterns were also reported by Karna et al^22^. in China, where linezolid (97.8%) and teicoplanin (95.6%) demonstrated the highest activity.

Vancomycin susceptibility in our isolates was relatively high (77.5%), whereas notable resistance was observed to tetracycline (58.3%), ciprofloxacin (51.7%), streptomycin (54.2%), and high-level gentamicin (43.3%). These findings align with Teffera et al^23^., who reported vancomycin susceptibility of 69.94% and equal ciprofloxacin susceptibility (50%), although gentamicin resistance was slightly higher (55.56%). A meta-analysis from Iran documented a 49.4% prevalence of high-level gentamicin resistance (HLGR)^24^, while Azzam et al^19^. reported a pooled HLGR rate of 61.1%.

In our study, gentamicin resistance was markedly higher among clinical isolates of *E. faecalis* (81.8%) and *E. faecium*(86.6%), consistent with Sobhanipoor et al^8^., who found resistance rates of 74% in clinical isolates compared with 18% in non-clinical ones. Ciprofloxacin resistance in *E. faecium* was also significantly elevated in clinical isolates (65.8%), whereas *E. faecalis* showed moderate resistance across all sources without significant variation (*p*> 0.5). These patterns correspond with observations by Gorski et al^15^. and Sobhanipoor et al^8^.. Clinical isolates also exhibited a higher prevalence of vancomycin resistance (66.7%), although the difference was not statistically significant. Similar trends were reported by Gorski et al^15^. and Sobhanipoor et al^8^., who noted that nearly all vancomycin-resistant isolates originated from clinical settings.

Recent evidence indicates that environmental exposure to biocides may contribute to the development of resistance through mechanisms such as intrinsic tolerance, phenotypic adaptation, acquisition of mobile genetic elements, chromosomal mutations, and biofilm-mediated protection^12^.

Despite the extensive use of biocides, comprehensive global data on bacterial susceptibility to them remain scarce. The lack of standardized testing protocols and the absence of defined interpretive breakpoints comparable to those used for antibiotics hinder meaningful comparison across studies. Additionally, many investigations assessing biocide susceptibility rely on relatively small sample sizes, further limiting generalizability^5^.

In this study, chlorhexidine (CHX) MIC values ranged from 0.5 to 16 µg/mL, with MIC₅₀ values of 4–8 µg/mL and an MIC<Subscript>90</Subscript> of 8 µg/mL. Clinical isolates of *E. faecalis* and *E. faecium*showed the highest proportions of elevated MICs (62.5% and 75% at 8 µg/mL, respectively), indicating reduced susceptibility. These findings are consistent with Kheljan et al^5^., who reported ECOFF values of 8 µg/mL for both species and noted that isolates with elevated CHX MICs were predominantly hospital-isolated. Pereira et al^25^. similarly reported CHX digluconate MICs ranging from 0.5 to 8 mg/L, with MIC₅₀ and MIC<Subscript>90</Subscript> values of 4 mg/L and 8 mg/L, respectively.

However, earlier studies documented substantially higher ECOFF thresholds of 64 µg/mL for *E. faecalis* and 32 µg/mL for *E. faecium*^26^. Sobhanipoor et al^8^. also reported markedly elevated CHX MICs in clinical isolates (16–128 µg/mL), while studies from Turkey and Spain documented MIC ranges of 6–64 µg/mL^27^. Gorski et al^15^. further observed that the highest CHX MIC<Subscript>90</Subscript> values originated from clinical samples. Pereira et al^28^. additionally noted that CHX MICs for clinical *E. faecalis* (4.8 mg/L) were comparable to those of environmental isolates.

For benzalkonium chloride (BCC), the MIC values among the *Enterococcal*isolates ranged from 2 to 32 µg/mL, with MIC₅₀ values between 4 and 16 µg/mL and an MIC<Subscript>90</Subscript> of 16 µg/mL. Clinical isolates demonstrated the highest proportion of elevated MICs, reflected in increased MIC₅₀ and MIC<Subscript>90</Subscript> values (16 µg/mL), indicating reduced susceptibility. Kheljan et al^5^. reported ECOFF thresholds of 16 µg/mL for *E. faecalis* and 32 µg/mL for *E. faecium*, and noted that isolates from healthy individuals exhibited the highest MIC<Subscript>90</Subscript> (16 µg/mL), a finding that contrasts with the pattern observed in our study. In contrast, Morrissey et al^26^. documented a lower ECOFF of 8 µg/mL for both species.

Sobhanipoor et al^8^. identified reduced BCC susceptibility at MIC values exceeding 8 µg/mL, occurring in 28% of clinical isolates and 52% of non-clinical isolates. These proportions were lower than those observed in our dataset, where 56.25% of *E. faecalis* and 53.6% of *E. faecium*clinical isolates exhibited MICs of 16 µg/mL. The higher frequency of reduced susceptibility to BCC among non-clinical isolates reported in earlier studies has been attributed to repeated exposure to this biocide in community environments. Continuous contact with low concentrations of BCC may facilitate the selection of tolerant strains, as suggested by Kheljan et al^5^. and Sobhanipoor et al^8^..

In the present study, isolates exhibiting resistance to ciprofloxacin, vancomycin, and gentamicin consistently demonstrated higher MIC₅₀ and MIC₉₀ values for both chlorhexidine (CHX) and benzalkonium chloride (BCC) compared with susceptible isolates. This pattern aligns with the findings of Gorski et al^15^., who reported elevated CHX MIC₅₀ and MIC₉₀ values among ciprofloxacin- and vancomycin-resistant *E. faecium*, indicating reduced CHX susceptibility. Similar associations between antibiotic resistance and increased CHX MICs have been documented by Sobhanipoor et al^8^. and Ignak et al^29^.. Alotaibi et al^27^. likewise observed that 95% of vancomycin-resistant and only 33% of vancomycin-susceptible *E. faecium* isolates from Danish hospitals exhibited a CHX MIC of 4 µg/mL.

With respect to BCC, Ignak et al^29^. reported higher MIC values among vancomycin-resistant *Enterococci*(VRE). Alotaibi et al^27^. also identified significant differences in susceptibility to BCC between VRE and vancomycin-susceptible *Enterococci*(VSE). In contrast, studies by Sobhanipoor et al^8^. and Suller et al^30^. found no significant MIC differences between VRE and VSE groups, which diverges from our observations.

Biofilm formation, a key virulence factor, was detected in 66.7% of isolates (*n* = 80), consistent with previous reports^31,32^. *E. faecalis* exhibited a higher prevalence of biofilm formation (71.4%) than *E. faecium*(61.4%). Clinical isolates of both species demonstrated the strongest biofilm-forming capacity, in agreement with Gorski et al^15^. and Son et al^33^.. Global studies similarly reported variable but generally higher biofilm prevalence in *E. faecalis*^34,35^.

Our results showed a decrease in biofilm formation in *Enterococci* at higher MIC levels, and a strong biofilm-producing isolates may paradoxically appear more susceptible to BCC as described by^15^. This phenomenon is often attributed to prior exposure to sub-inhibitory (sub-MIC) concentrations of antimicrobials, which can alter cell physiology, reduce biofilm biomass, or modify surface structures. Sub-MIC exposure may therefore influence both biofilm behavior and susceptibility profiles, producing patterns that cannot be explained solely by intrinsic resistance mechanisms^36^.

ATP-binding cassette (ABC) transporters play a key role in mediating multidrug resistance in *Enterococci*. Co-expression of the *efrA* and *efrB* genes results in the formation of the heterodimeric *EfrAB* transporter, which contributes to antimicrobial resistance^37^. In the present study, the *efrAB* gene was detected in 54% of *E. faecalis* and 53% of *E. faecium*isolates, yielding an overall prevalence of 50.8%, a pattern closely aligned with the findings of Sobhanipoor et al^8^.. Mirzaii et al^38^. similarly reported the presence of *efrAB* in 51% of *E. faecium*isolates. In contrast, Shiadeh et al^37^. identified *efrA* and *efrB* in all *E. faecalis*isolates, and higher prevalence rates have also been documented in earlier studies by Valenzuela et al^39^. and Kang et al^40^..

The *emeA* gene encodes an efflux pump located on a transmissible genetic element within the *Enterococcus* chromosome^5,41^. In the present study, *emeA* was detected in 39.7% of *E. faecalis* isolates and 28% of *E. faecium*isolates, yielding an overall prevalence of 34.2%. These findings closely align with those of Kheljan et al^5^., who reported *emeA* in 42.8% of *E. faecalis* and 27.8% of *E. faecium*isolates. In contrast, Panthee et al^42^. documented the presence of *emeA* in all *E. faecalis* isolates examined.

In our analysis, both *efrAB* and *emeA*genes were significantly more common in clinical isolates compared to environmental and healthy carrier ones. This pattern corroborates the observations of Sobhanipoor et al^8^., who reported *emeA* in 57.4% of clinical isolates compared with 32% of non-clinical isolates, and *efrAB* in 66.7% and 40% of clinical and non-clinical isolates, respectively.

In our study, isolates carrying either *efrAB* or *emeA*exhibited markedly higher rates of gentamicin resistance (86.5% and 53.8%, respectively) compared with gene-negative isolates. This observation aligns with Sobhanipoor et al^8^., who reported that both genes were present in all high-level gentamicin-resistant (HLGR) isolates. Lerma et al^43^. similarly demonstrated that exposure to gentamicin induces upregulation of *efrAB*, further supporting the association between these efflux systems and aminoglycoside resistance. In contrast, our findings revealed no relationship between ciprofloxacin resistance and the presence of efflux pump genes, consistent with the results of Sobhanipoor et al^8^..

In our dataset, no significant association was identified between the MIC values of chlorhexidine (CHX) or benzalkonium chloride (BCC) and the presence of *efrAB* or *emeA* despite higher MIC₉₀ values observed in *efrAB*-harbouring isolates. This differs from Sobhanipoor et al^8^., who reported that these genes were significantly linked to reduced CHX susceptibility but not to BCC. Lerma et al^43^. also noted that all *E. faecalis* isolates with decreased CHX susceptibility harbored *efrAB*, whereas only 12% of *E. faecium* isolates with reduced susceptibility carried the gene. In agreement with our results, previous studies found no difference in CHX susceptibility between *emeA*-positive and *emeA*-negative isolates^5,44^.

Our findings further demonstrated that isolates harboring *emeA*, *efrAB*, or both genes exhibited higher MIC₉₀ values for biocides compared with gene-negative isolates. Isolates lacking these genes consistently showed lower MIC₉₀ values, regardless of their phenotypic profile, a pattern also reported by Kheljan et al^5^.. However, several studies have emphasized that the presence of efflux-pump-associated genes does not always correlate with reduced biocide susceptibility^45,46^. Despite this, our results indicate that isolates carrying at least one efflux-pump gene tended to display higher MIC₉₀ values than those without such genes.

## Study limitations

This study is limited by the use of planktonic MIC assays that do not capture biofilm-associated susceptibility, the absence of functional or expression analysis of efflux pump genes, and a relatively small sample size that may have reduced statistical power. Additionally, the study population was limited to a single setting, which may affect generalizability.

## Conclusion

our study revealed that clinical *Enterococcus* isolates displayed heightened antimicrobial resistance, enhanced biofilm-forming capacity, and elevated CHX and BCC MICs relative to environmental and commensal isolates. The strong association between efflux pump genes (*efrAB* & *emeA)* and both gentamicin resistance and increased biocide MICs underscores the role of efflux-mediated mechanisms in reduced biocide susceptibility and could contribute to the development of cross-resistance phenotypes. These findings highlight the importance of considering both phenotypic and genotypic factors, including biofilm formation and efflux pump genes, when interpreting MIC-based susceptibility data.

## Electronic Supplementary Material

Below is the link to the electronic supplementary material.

Supplementary Material 1

## Data Availability

The datasets generated and/or analyzed during the current study are available from the corresponding author upon reasonable request.

## Abbreviations

CLSI: Clinical and Laboratory Standards Institute
MIC: Minimum inhibitory concentration
ECOFF: Epidemiological cut-off
MDR: multidrug-resistant
CBs: Cationic biocides
QACs: quaternary ammonium compounds

## Declarations

### Supplementary Information

Supplementary Table 1: Antimicrobial resistance profiles of the studied *Enterococcus* isolates identified by the Vitek-2 system (*n* = 120). Supplementary Table 2: Association between CHX and BCC MIC levels and susceptibility to ciprofloxacin, vancomycin, and gentamicin. Supplementary Table 3: Biofilm-forming strength of *E. faecalis* and *E. faecium* isolated from different sources.Supplementary Table 4: Frequency distribution of the Efflux-pump genes among *E. faecalis* and *E. faecium* isolates. Supplementary Table 5: Association between Chlorhexidine digluconate and Benzalkonium chloride MICs with the presence of efflux-pump Genes. Supplementary Fig. 1: PCR Gel Electrophoresis of the amplified product of *emeA*, and *efrB* genes. Supplementary Fig. 2: PCR Gel Electrophoresis of the amplified product of *efrA* gene.
