# Supplementary material for: Cationic biocide susceptibility and tolerance-associated genes in clinical, environmental, and commensal Enterococcus isolates
Source: Sci Rep. 2026 May 13;16:15063. doi: 10.1038/s41598-026-51663-z (PMC13172038; doi:10.1038/s41598-026-51663-z)
Supplement: Supplementary file 1 — Supplementary Material 1 [file 41598_2026_51663_MOESM1_ESM.docx]

**Supplementary Tables and Figures**

**Supplementary Fig. S1. PCR Gel Electrophoresis of the amplified product of *emeA*, and *efrB* genes**


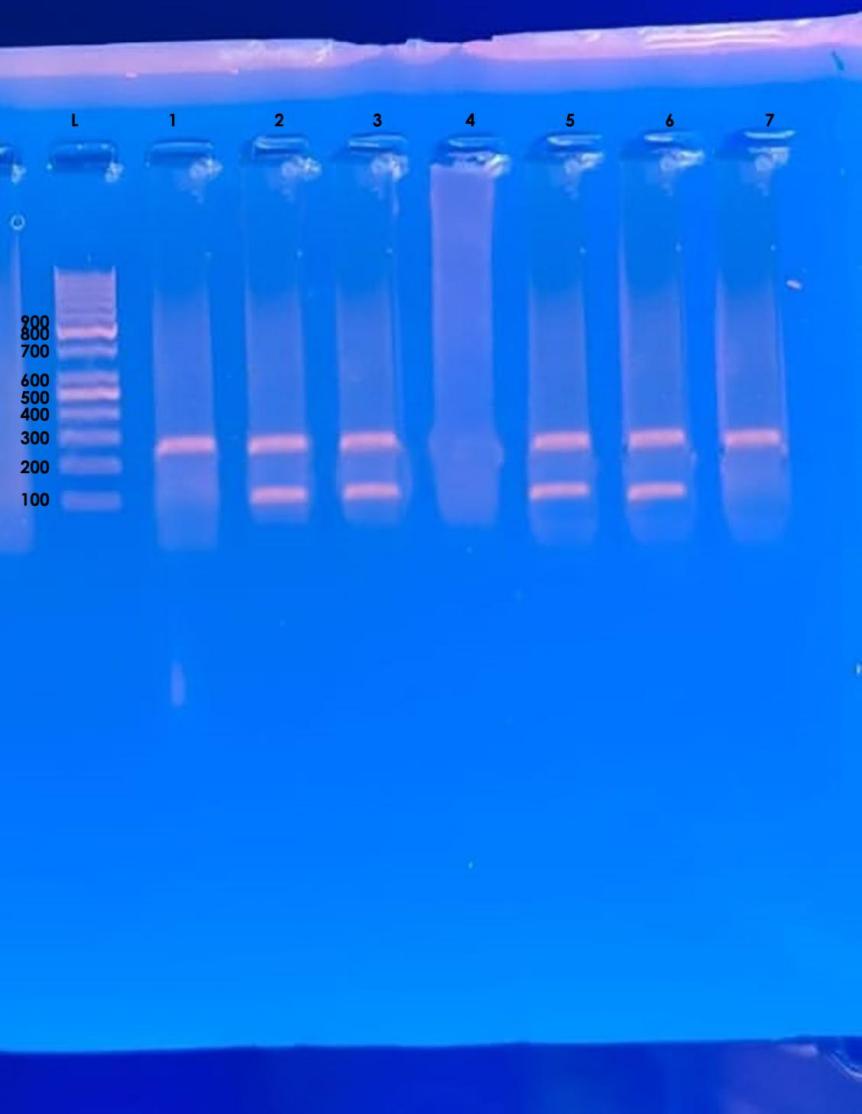


Lane L: DNA Molecular Size ladder (100−1000 bp). Lanes 2, 3,5, and 6: Positive for *emeA* with a band size of 128 bp. Lanes 1,4, and 7: Negative for *emeA*. Lanes 1-3, and 5- 7: Positive for *efrB* with a band size of 211 bp. Lane 4: Negative for *efrB.*

**Supplementary Fig. S2. PCR Gel Electrophoresis of the amplified product of *efrA* gene.**


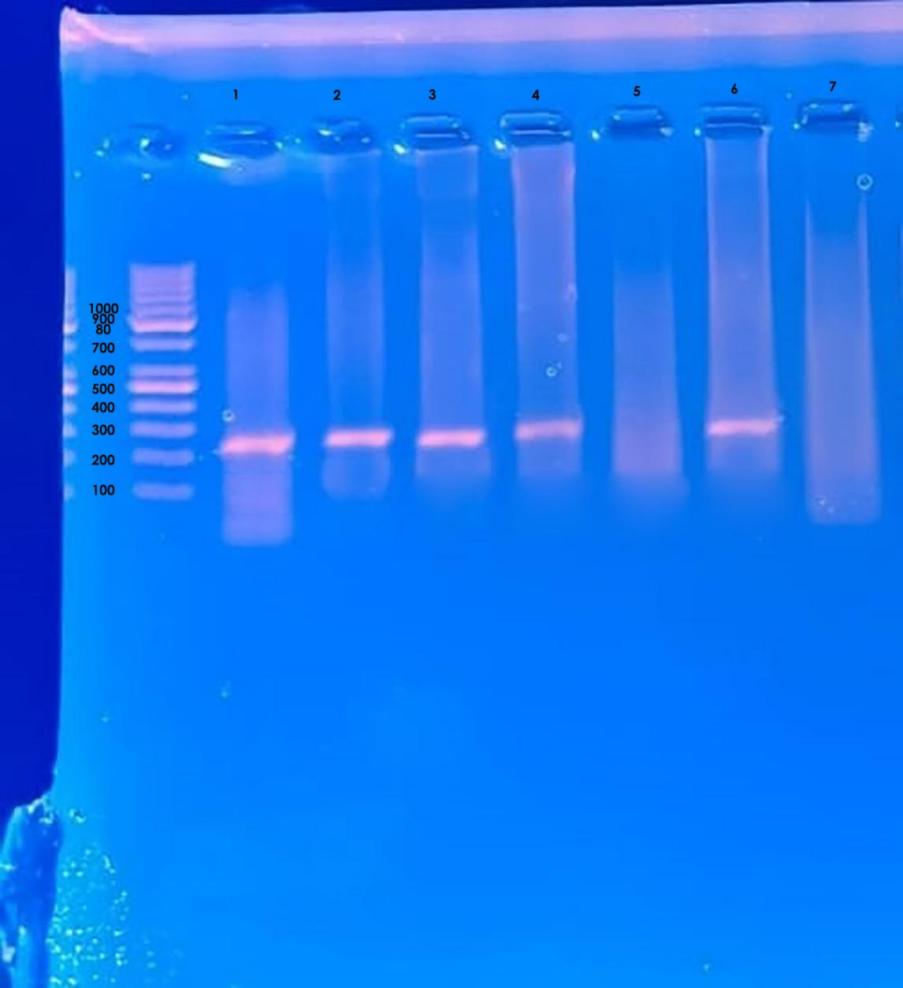


Lane L: DNA Molecular Size ladder (100−1000 bp). Lanes 1-4 and 6: Positive for *efrA* with a band size of 224 bp. Lanes 5 and 7: Negative for *efrA.*

**Supplementary Table S1. Antimicrobial resistance profiles of the studied *Enterococcus* isolates identified by the Vitek‑2 system (n = 120).**

| **Antibiotic Group** | **Antimicrobial Agents** | ***Enterococcus* spp.**  **MIC Breakpoints (*CLSI, 2025*)** | | |
| --- | --- | --- | --- | --- |
|  |  | **Sensitive** | **Intermediate** | **Resistant** |
|  |  | **N (%)** | **N (%)** | **N (%)** |
| **Penicillin** | Ampicillin | 65 (54.2%) | 0 (0%) | 55(45.8%) |
| **Aminoglycosides** | Gentamycin high level | 68 (56.7%) | 0 (0%) | 52 (43.3%) |
|  | Streptomycin high level | 55 (45.8%) | 0 (0%) | 65 (54.2%) |
| **Macrolides** | Erythromycin | 38 (31.7%) | 30 (25%) | 52 (43.3%) |
| **Fluoroquinolones** | Ciprofloxacin | 58 (48.3%) | 0 (0%) | 62 (51.7%) |
| **Glycopeptides** | Vancomycin | 93 (77.5%) | 0 (0%) | 27 (22.5%) |
| **Oxazolidinones** | Linezolid | 88 (87.5%) | 0 (0%) | 15 (12.5%) |
| **Lipoglycopeptides** | Teicoplanin | 100 (83.3%) | 1 (1%) | 19 (15.8%) |
| **Tetracyclines** | Tetracycline | 50 (41.7 %) | 0 (0%) | 70 (58.3%) |
| **Glycycline** | Tigecycline | 120 (100%) | 0 (0%) | 0 (0%) |

**Supplementary Table S2. Association between CHX and BCC MIC Levels and Susceptibility to ciprofloxacin, vancomycin, and gentamicin**

| 1. ***faecalis* N=63** | | **CHX MIC50 (µg/mL)** | **CHX MIC90 (µg/mL)** | **BCC MIC50 (µg/mL)** | **BCC MIC90 (µg/mL)** |
| --- | --- | --- | --- | --- | --- |
| **CIP** | **R (n=24 )** | 8 | 8 | 8 | **16** |
|  | **S (n=39 )** | 4 | 8 | 4 | **8** |
| **VAN** | **R (n=9 )** | **8** | **8** | **8** | **32** |
|  | **S (n= 54)** | **4** | **8** | **8** | **16** |
| **GEN** | **R (n= 22)** | **8** | **8** | **8** | **16** |
|  | **S (n= 41)** | **4** | **8** | **4** | **8** |
| 1. ***faecium* N=57** | | **CHX MIC50 (µg/mL)** | **CHX MIC90 (µg/mL)** | **BCC MIC50 (µg/mL)** | **BCC MIC90 (µg/mL)** |
| **CIP** | **R (n=38)** | **8** | **8** | **4** | 16 |
|  | **S (n=19 )** | **4** | **8** | **2** | 8 |
| **VAN** | **R (n=18 )** | **8** | **16** | **8** | 16 |
|  | **S (n= 39)** | **4** | **8** | **8** | 8 |
| **GEN** | **R (n= 30)** | **4** | **8** | **4** | 16 |
|  | **S (n= 27)** | **4** | **8** | **4** | 16 |

**Supplementary Table S3. Biofilm-forming strength of *E. faecalis* and *E. faecium* isolated from different sources.**

| **Biofilm strength** | ***E. Faecalis* (n=63)** | | | | ***E. Faecium* (n=57)** | | | |
| --- | --- | --- | --- | --- | --- | --- | --- | --- |
|  | **Clinical (n=32)** | **Surfaces (n=16)** | **Healthy volunteers (n=15)** | **Total** | **Clinical (n=28)** | **Surfaces (n=17)** | **Healthy volunteers(n=12)** | **Total** |
|  | **N %** | **N %** | **N %** | **N %** | **N %** | **N %** | **N %** | **N %** |
| **No biofilm (I)** | 1 3.125 | 6 37.5 | 11 73.3 | 18 28.6 | 7 25 | 8 47 | 7 58.3 | 22 38.6 |
| **Weak (II)** | 2 6.25 | 0 0.00 | 0 0.00 | 2 3.2 | 1 3.6 | 0 0.00 | 0 0.00 | 1 1.8 |
| **Moderate (III)** | 7 21.9 | 5 31.25 | 1 6.7 | 13 20.6 | 3 10.7 | 2 11.8 | 0 0.00 | 5 8.8 |
| **Strong (IV)** | 22 68.75 | 5 31.25 | 3 20 | 30 47.6 | 17 60.7 | 7 41.2 | 5 41.7 | 29 50.8 |
| **Total biofilm producers (II, III,IV)** | 31 96.9 | 10 62.5 | 4 26.7 | 45 71.4 | 21 75 | 9 53 | 5 41.7 | 35 61.4 |
| **p-value** | **P1 < 0.001∗** | | | | **P2 <0.001∗** | | | |

P1: p value for comparing between the 3 sources of *E.faecalis* regarding biofilm-forming strength.

P2: p value for comparing between the 3 sources of *E.faecium* regarding biofilm-forming strength.

∗Statistically signifcant at p ≤ 0.05.

**Supplementary Table S4. Frequency distribution of the Efflux-pump genes among *E. faecalis* and *E. faecium* isolates.**

| **Variables** | | ***E. faecalis***  **(n=63)** | | ***E. faecium***  **(n=57)** | | | **Total**  **(n=120)** | | **Test of significance** | | **p*** |
| --- | --- | --- | --- | --- | --- | --- | --- | --- | --- | --- | --- |
|  |  | **No.** | **%** | **No.** | **%** | | **No.** | **%** |  |  |  |
| ***efrAB*** | | | | | | | | | | | |
| **Present**  **Absent** | | 34  29 | 54  46 | 27  30 | 53  47 | | 61  59 | 50.8  49.2 | 0.52 | | 0.47 |
| ***EmeA*** | | | | | | | | | | | |
| **Present**  **Absent** | | 25  38 | 39.7  60.3 | 16  41 | 28  72 | | 41  79 | 34.2  65.8 | 1.7 | | 0.18 |
| **Co-existence of *efrAB+EmeA*** | | | | | | | | | | | |
| **Present**  **Absent** | 17  46 | | 27  73 | 10  47 | | 17.5  82.5 | 27  93 | 22.5  77.5 | | 1.52 | 0.216 |

P: p value for comparing between the *E. faecalis* and *E. faecium* isolates regarding efflux-pump genes carriage.

∗Statistically signifcant at p ≤ 0.05.

**Supplementary Table S5. Association between Chlorhexidine digluconate and Benzalkonium chloride MICs with the presence of efflux-pump Genes**

| **Biocide Associated Genes** | | **Chlorhexidine digluconate MIC (μg/mL)** | | | | | | | | | | | | | | | | | | | | | | | | | | | | | **P** | |
| --- | --- | --- | --- | --- | --- | --- | --- | --- | --- | --- | --- | --- | --- | --- | --- | --- | --- | --- | --- | --- | --- | --- | --- | --- | --- | --- | --- | --- | --- | --- | --- | --- |
|  |  | **Total** | **0.5** | | | | | **1** | | | | | **2** | | | | **4** | | | | | **8** | | | | | **16** | | | |  |  |
|  |  |  | **NO** | | **%** | | **NO** | | | **%** | | **NO** | | | **%** | **NO** | | | **%** | | **NO** | | | **%** | | **NO** | | | **%** | |  |  |
| ***efrAB***  positive negative | | **61**  **59** | 0 (0.0)  1 1.7 | | | | | 1 1.6  4 6.8 | | | | | 7 11.7  11 18.6 | | | | 20 32.8  20 33.9 | | | | | 32 52.5  23 40.4 | | | | | 1 1.6  0 (0.0) | | | | **0.29** | |
| ***emeA***  positive negative | | **41**  **79** | 0 (0.0)  1 1.3 | | | | | 1 2.4  4 5.1 | | | | | 9 22  9 11.4 | | | | 15 36.6  25 31.6 | | | | | 16 39  39 49.4 | | | | | 0 (0.0)  1 1.3 | | | | **0.5** | |
| **Biocide Resistance Genes** | **Total** | | | **Benzalkonium chloride MIC (μg/mL)** | | | | | | | | | | | | | | | | | | | | | | | | | | **P** | |  |
|  |  |  |  | **2** | | | | | **4** | | | | | **8** | | | | | | **16** | | | | | **32** | | | | |  |  |  |
|  |  |  |  | **NO** | | **%** | | | **NO** | | **%** | | | **NO** | | | | **%** | | **NO** | | | **%** | | **NO** | | | **%** | |  |  |  |
| ***efrAB*** positive  negative | **61**  **59** | | | 2 3.3  4 6.8 | | | | | 6 9.8  13 22 | | | | | 24 39.3  15 25.4 | | | | | | 28 46  25 42.4 | | | | | 1 1.6  2 3.4 | | | | | **0.21** | |  |
| ***emeA***  positive  negative | **41**  **79** | | | 1 2.4  5 6.3 | | | | | 5 12.2  14 17.7 | | | | | 15 36.6  24 30.4 | | | | | | 17 41.5  36 45.6 | | | | | 2 4.9  1 1.3 | | | | | **0.52** | |  |

P: p value for comparing between the variable CHX and BCC MICs values regarding acquisition of efflux-pump genes

∗Statistically signifcant at p ≤ 0.05.
